# Qualitative Individual Differences are Useful, but Reliability Should be Assessed and Not Assumed

**DOI:** 10.5334/joc.169

**Published:** 2021-08-27

**Authors:** Craig Hedge

**Affiliations:** 1 Aston University, School of Psychology, College of Health & Life Sciences, Birmingham, UK; 2 Cardiff University, School of Psychology, Cardiff, UK

**Keywords:** Statistical analysis, Cognitive Control, Mathematical modelling

## Abstract

Rouder and Haaf (2021) propose that studying qualitative individual differences would be a useful tool for researchers. I agree with their central message. I use this commentary to highlight examples from the literature where similar questions have been asked, and how researchers have addressed them with existing tools. I also observe that while the hierarchical Bayesian framework is a useful tool for studying individual differences, it does not relieve us of the requirement to evaluate the forms of reliability that are critical to our research questions.

Rouder and Haaf (2021) outline an approach in which qualitative variations in experimental effects can be a valuable tool to researchers, by constraining theory and leading us to questions about individual differences that we might not reach otherwise. I am keen to see this approach embraced and hope this commentary will contribute to the discussion on where and how it can be best used.

## Do researchers think about qualitative individual differences?

Rouder and Haaf ask whether the identification of qualitative individual differences speaks to researchers. Indeed, I think the ‘does everybody…’ question formalises intuitions that I and colleagues have had about our data. This prompted me to consider how I would previously have tackled these kinds of questions.

In the discussion of a recent paper examining individual differences in strategic changes in the speed-accuracy trade-off (SAT), I reflected on whether every individual responds to the instruction to prioritise speed over accuracy in the same way (Hedge et al., 2019). We typically assume that the SAT reflects a change in how much evidence an individual requires to make a decision, though it may also involve changes in how evidence is processed (Rae et al., 2014). From this I speculated ‘*does everybody* change in more than just their strategy?’. I ran an analysis for one dataset here to illustrate what I would do with my existing toolkit.^1^ I fit multiple variants of a cognitive model to each participant’s data separately. I then used model comparisons to determine the most parsimonious account for each person. One model assumed that participants only changed their threshold for how much evidence is required, which was sufficient to explain the data of 10 out of 81 participants. A second model assumed that the duration of perceptual and motor processing changed in addition to their threshold (47/81). A third further assumed that the efficiency of evidence processing was affected (24/81). Based on this, I would conclude that there are qualitative individual differences in the speed-accuracy trade-off.

I also see qualitative individual differences assumed or implied in the literature. Responder analyses are common in clinical studies and are sometimes used in other areas (e.g. working memory training, see Tidwell et al., 2014). There, researchers define a cut-off that represents a clinically significant effect, and then may ask what covaries with the presence or absence of that effect. Finally, while Haaf and Rouder (2017) have shown that “everybody Stroops”, perhaps not everybody spider-Stroops (Watts et al., 1986). Using a mixed ANOVA, Watts et al. show that spider related words interfere with colour naming for individuals with a spider phobia to a greater extent than controls. They also show that the distribution of observed interference scores is centred close to zero for controls.

The questions in these examples do not take the same form as the ones posed by Rouder and Haaf. The model comparisons in the speed-accuracy trade-off example do not ask about the direction of changes or formally test whether qualitative individual differences are present at the sample level. In the spider-Stroop example, testing for an interaction in an ANOVA does not ask whether some individuals show null or negative (true) effects. However, these existing approaches may be a barrier to the uptake of new tools if they scratch the same theoretical itch for researchers.

## Are estimated true effects reliable/stable?

A desirable property of the hierarchical Bayesian approach proposed by Rouder and Haaf is the ability to estimate ‘true’ effects from observed effects by incorporating assumptions about the magnitude of trial noise. There has been recent concern that traditionally measured experimental effects taken from widely used tasks are not as reliable as we would like them to be for asking questions about individual differences (e.g. Hedge et al., 2018; Parsons et al., 2019). The same concern applies to qualitative individual differences in principle – it is difficult to identify factors that covary with the presence or absence of an effect if an individual sometimes shows an effect and sometimes does not. Do hierarchical models relieve us of this concern?

The idea of true scores is associated with classical test theory (Novick, 1966), where it is assumed that observed measures reflect variation in people’s true values on the dimension of interest plus some measurement error. When we assess reliability, we estimate the ratio of signal (variation in true scores) to noise (error) by examining the consistency of participants’ scores over some form of repeated measurement. It has been stated that reliability is not a property of tasks or procedures, but rather a property of a set of scores obtained from a given population (Wilkinson, 1999). This is because the magnitude of the signal and the noise are context dependent (Cronbach et al., 1963). For example, there may be more variance in true Stroop effects in a clinical population than in a healthy population. Measurement error is also potentially comprised of multiple sources. I agree with Rouder and Haaf that trial noise is a large component in reaction time-based effects (see Supplementary Material D of Hedge et al., 2018), though there can be additional sources of error in a test-retest reliability context (e.g. fluctuations in mood or health). Until we have assessed the test-retest reliability of a task in our population, we do not know if our qualitative or quantitative differences reflect stable characteristics of those individuals.

**Figure 1:**
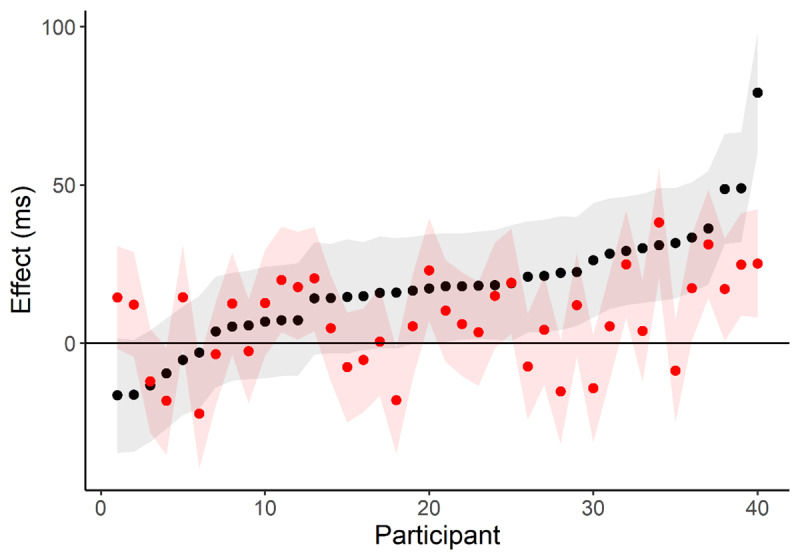
Estimated SNARC effects from session 1 (black) and session 2 (red) from Hedge et al., (2018). Shaded regions are 95% credible intervals. Estimates are sorted by their size in session 1.

As an illustration, I applied Rouder and Haaf’s quid() function to test-retest reliability data for the spatial-numeric association of response codes (SNARC) effect from Hedge et al. (2018).^2^ I chose this task because the effect in mean reaction times was relatively small (15ms and 8ms in sessions one and two respectively), so it is likely that there are qualitative individual differences. I applied the analysis to session one (***Figure 1***; black) and session two (red) separately, to highlight the conclusion we would draw if we only had data from a single session. We would reach the same conclusion about whether qualitative individual differences are present from both sessions – Bayes factors favour the unconstrained model. However, if we were to ask “what kind of person shows a positive/null/negative SNARC effect”, then we might select different individuals at different time points depending on how we identify them. Twelve out of forty participants have numerically positive effects in one session and negative effects in the other. Further, the 95% credible interval for twelve participants includes zero in one session and not the other.

Several papers have shown that hierarchical Bayesian models can improve our estimates of individual differences (Brown et al., 2020; Rouder & Haaf, 2019; Wiecki et al., 2013), and the illustration above does not contradict this. The key point is that we still need to evaluate the forms of reliability that are important to our research questions to be able to make appropriate generalisations about qualitative individual differences.

## Footnotes

Data are the random-dot motion task data from Hedge et al. (2019). The script and model outputs for this new analysis are on the OSF (*https://osf.io/yh4ak/*).

R code for this analysis can be found on the Open Science Framework.
